# Stability of petal color polymorphism: the significance of anthocyanin accumulation in photosynthetic tissues

**DOI:** 10.1186/s12870-019-2082-6

**Published:** 2019-11-14

**Authors:** José Carlos Del Valle, Cristina Alcalde-Eon, Mª. Teresa Escribano-Bailón, Mª. Luisa Buide, Justen B. Whittall, Eduardo Narbona

**Affiliations:** 1 Department of Molecular Biology and Biochemical Engineering, Pablo de Olavide University, 41013 Seville, Spain; 2 Grupo de Investigación en Polifenoles (GIP), University of Salamanca, 37007 Salamanca, Spain; 3 Department of Biology, Santa Clara University, Santa Clara, CA 95053 USA

**Keywords:** Anthocyanins, Flavonoids, Flower color polymorphism, Loss of pigmentation, Non-pollinator mediated selection, Plant secondary metabolites, Pleiotropy

## Abstract

**Background:**

Anthocyanins are the primary source of colour in flowers and also accumulate in vegetative tissues, where they have multiple protective roles traditionally attributed to early compounds of the metabolic pathway (flavonols, flavones, etc.). Petal-specific loss of anthocyanins in petals allows plants to escape from the negative pleiotropic effects of flavonoid and anthocyanins loss in vegetative organs, where they perform a plethora of essential functions. Herein, we investigate the degree of pleiotropy at the biochemical scale in a pink-white flower colour polymorphism in the shore campion, *Silene littorea*. We report the frequencies of pink and white individuals across 21 populations and underlying biochemical profiles of three flower colour variants: anthocyanins present in all tissues (pink petals), petal-specific loss of anthocyanins (white petals), and loss of anthocyanins in all tissues (white petals).

**Results:**

Individuals lacking anthocyanins only in petals represent a stable polymorphism in two populations at the northern edge of the species range (mean frequency 8–21%). Whereas, individuals lacking anthocyanins in the whole plant were found across the species range, yet always at very low frequencies (< 1%). Biochemically, the flavonoids detected were anthocyanins and flavones; in pigmented individuals, concentrations of flavones were 14–56× higher than anthocyanins across tissues with differences of > 100× detected in leaves. Loss of anthocyanin pigmentation, either in petals or in the whole plant, does not influence the ability of these phenotypes to synthesize flavones, and this pattern was congruent among all sampled populations.

**Conclusions:**

We found that all colour variants showed similar flavone profiles, either in petals or in the whole plant, and only the flower colour variant with anthocyanins in photosynthetic tissues persists as a stable flower colour polymorphism. These findings suggest that anthocyanins in photosynthetic tissues, not flavonoid intermediates, are the targets of non-pollinator mediated selection.

## Background

Mutations are the primary source of genetic variation in all organisms and have a key contribution to phenotypic diversity [1, 2], but not all mutations are evolutionarily relevant. Some phenotypic changes are produced through spontaneous mutations with deleterious effects that are consistently eliminated by purifying selection [3]. In contrast, persistent phenotypic changes arise from mutations maintained by balancing selection through frequency-dependent or heterogeneous selection or through the promotion of multiple adaptive peaks [4–8], resulting in a population polymorphism for that trait [9–11]. Factors that may determine why some new phenotypes are fleeting and some persists as polymorphisms are still open [12, 13], but the study of flower color is helping to shed light to this issue [14–17].

Flower color variation has drawn the attention of many naturalists through the history [18–20], and nowadays continues to be an important focus of research to evolutionary biologist [14, 21]. Flower color has been considered as an adaptive trait for pollinator attraction [22], but underlying pigments also have other functions, especially in vegetative tissues. Anthocyanins are the most common plant pigment that color flowers, conferring orange, red, pink and blue colors [23] that attract diverse functional groups of pollinators [22, 24]. For example, variation in anthocyanin content in monkeyflowers (*Mimulus*) results in red and pink-flowered species that are visited by hummingbirds and bees, respectively [25, 26]. In vegetative tissues, anthocyanins may show protective roles such as sunscreens, antioxidants or antipathogens, among others [27, 28]. Thus, loss of anthocyanins may affect pollinator activity, but may also have physiological effects depending on whether they are accumulated or not in vegetative tissues. If the loss of anthocyanins is confined to the flowers (usually in petals), the rest of the plant can produce anthocyanins and reduce any negative pleiotropic effects in other tissues [29, 30], whereas anthocyanins-lacking individuals in the whole plant potentially grow and reproduce, but frequently exhibit fitness disadvantages that seem to explain their scarcity in the wild [31–33] (see Additional file 1: Table S1).

Petal-specific loss of anthocyanins is frequently induced by regulatory mutations (that is changes in the regulation of gene expression) and shows a mutation bias to Myb transcription factors, the key regulatory factors controlling anthocyanin biosynthesis in plants [30, 34]. Different copies of Myb proteins regulate floral and vegetative anthocyanins, thus the specificity of this regulation is predicted to have low pleiotropic consequences [35]. For example, in *Ipomoea purpurea* mutations the regulatory *IpMyb1* gene are responsible for anthocyanin loss in pigmented flowers [36]. However, these mutations do not affect the fitness of white-flowered plants and show equal or even higher reproductive success than that of the pigmented individuals [15, 29].

Anthocyanin-lacking individuals, on the other hand, are conferred by loss-of-function in any of the structural loci or whole plant regulatory genes of the anthocyanin biosynthetic pathway (hereafter ABP) [30, 34]. Loss-of-function mutations may target a high spectrum of genes since there are more possible loci that could confer the non-pigmented phenotype. Thus, the inactivation of any structural gene of the pathway often limit the flux down the ABP and block the anthocyanin production, but also may affect the synthesis of uncolored/pale-yellow non-anthocyanin flavonoids in the side branches of the pathway [23]. These flavonoids, such as flavones or flavonols, also perform important ecological functions because they show similar or even more protective functions against environmental stressors than anthocyanins themselves [37]. Therefore, the persistence of loss-of-function mutations should be limited by the negative pleiotropic effects associated to the absence of anthocyanins and/or intermediate non-anthocyanin flavonoids [14, 17, 38]. The selection against these variants may depend on where the mutation occurs and the associated negative consequences for the flavonoid loss.

Loss of pigmentation, particularly due to absence of anthocyanins, represents the most frequent cases of flower color polymorphism in plants [39, 40]. White-flowered morphs represent valuable natural genotypes to know the possible selective disadvantages of lack of anthocyanins in the whole plant [33, 39, 41–43], but the non-anthocyanin flavonoid composition of such plants is unknown. In addition, the quantities of anthocyanins are usually correlated with those of non-anthocyanin flavonoids, at least in some tissues, and the concentrations of the latter are even higher than that of anthocyanins [44, 45]. Consequently, it is difficult to distinguish which group of flavonoids is responsible for the putative selective disadvantage of anthocyanin lacking plants [31–33], and studies that clearly differentiate between flavonoid groups are limited.

The shore campion *Silene littorea* Brot. (Caryophyllaceae) is an annual pink-flowered species that accumulates anthocyanins and non-anthocyanin flavonoids in petals and in calyces, leaves and stems [44, 46]. The accumulation of both kinds of flavonoids in vegetative tissues is highly variable and seems to respond to light stress [46]. *Silene littorea* grows along the Iberian coast, and exhibits an anthocyanin-based pink-white flower color polymorphism in two populations of the northwest distribution range, but anthocyanin-lacking individuals are occasionally observed in some populations [47]. In *S. littorea*, petal-specific polymorphism is likely due to downregulation of the flavanone-3-hydroxylase (*F3 h*) gene through a downregulation of the *SlMyb1a* transcription factor [47], but genetic causes of anthocyanin-lacking plants are still unknown. Flowers of this species are mainly visited by generalist pollinators from the orders Diptera, Hymenoptera, and Lepidoptera; however, they do not seem to show strong pollinator preference for either pink or white flowers (M.L.B. 2019, unpublished data). Thus, the occurrence of petal anthocyanin loss and whole-plant anthocyanin loss individuals in *S. littorea* (hereafter PAL and WAL phenotypes, respectively) offers an excellent opportunity for understanding the importance of non-pollinator selection due to lack of anthocyanins and/or non-anthocyanin flavonoids.

In this study, we seek to understand the factors that determine the fate of different forms of anthocyanin variation in *S. littorea*. Thus, we investigated the population frequency of three anthocyanin phenotypes (PAL, WAL and fully pigmented) in 21 populations across the species distribution range over five years. Then, we used high-performance liquid chromatography coupled with diode-array detection and electrospray ionization tandem mass spectrometry (HPLC-DAD-MS^n^) to study the flavonoid profiles at the whole plant level in the fully pigmented phenotype and compare them to that of white-flowered variants (i.e. PAL and WAL phenotypes); after that, this study was expanded to more individuals and populations using spectrophotometric quantification of flavonoids. Because of the negative consequences of the absence of flavonoids [30, 38], we expect the PAL phenotype to be more common within a population compared to WAL. Thus, loss of anthocyanins and non-anthocyanin flavonoids are expected to be limited to petals in PAL plants, but extended to the whole plant in WAL individuals.

## Results

### Frequency of PAL, WAL and fully pigmented phenotypes

**Fig. 1:**
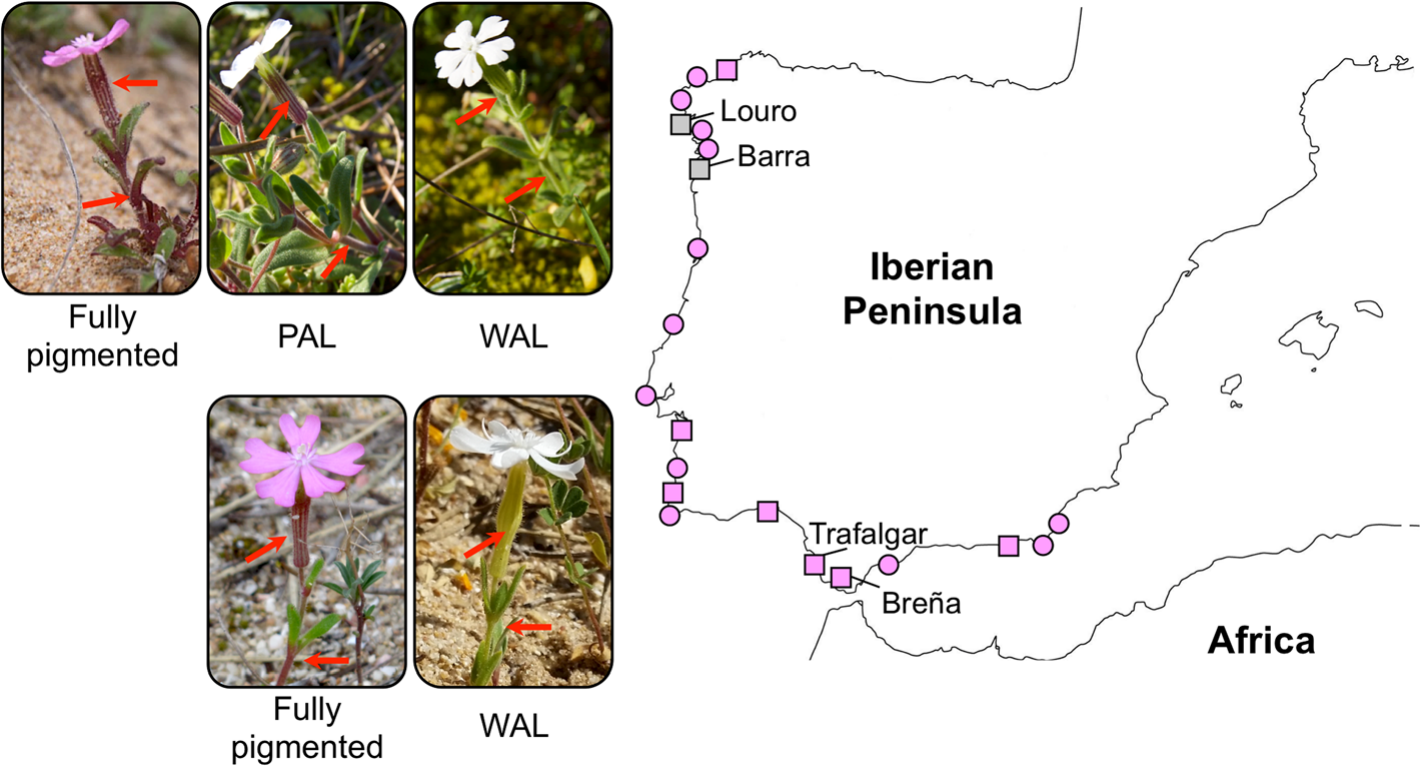
*Silene littorea* sampling and phenotypes with respect to anthocyanin accumulation. The map shows 21 populations covering the distribution range of *S. littorea* where frequencies of petal anthocyanin loss (PAL) and whole-plant anthocyanin loss (WAL) phenotypes were estimated (see Additional file 2: Table S2). Pink circles indicate populations in which only fully pigmented individuals (pink petals and anthocyanic photosynthetic tissues) are found, pink squares show populations in which WAL individuals are also found in at least one year of the studied period, and white squares represent populations where WAL and PAL individuals are found. The two polymorphic populations (Louro and Barra) and the two non-polymorphic populations (Trafalgar and Breña) in which flavonoids were biochemically analyzed are named. Photographs of the three phenotypes present in polymorphic (above) and non-polymorphic populations (below) are presented. Note the differences in anthocyanin accumulation in calyces and stems in fully pigmented and PAL plants vs. WAL individuals (indicated by red arrows) (more photographs available in Additional file 3: Figure S1)

Our population surveys confirmed that the PAL phenotype is limited to two populations in the northern portion of the species range (Fig. 1). A polymorphism from 8 to 21% of PAL plants has been maintained over the years at these two populations (Additional file 2: Table S2). In contrast, WAL individuals were found in nine of the 21 populations surveyed, including the two polymorphic populations, but always at very low frequencies (< 1% of total plants; Fig. 1 and Additional file 2: Table S2) and without any clear geographic pattern.

### Flavonoid identification and composition in each plant tissue

**Fig. 2:**
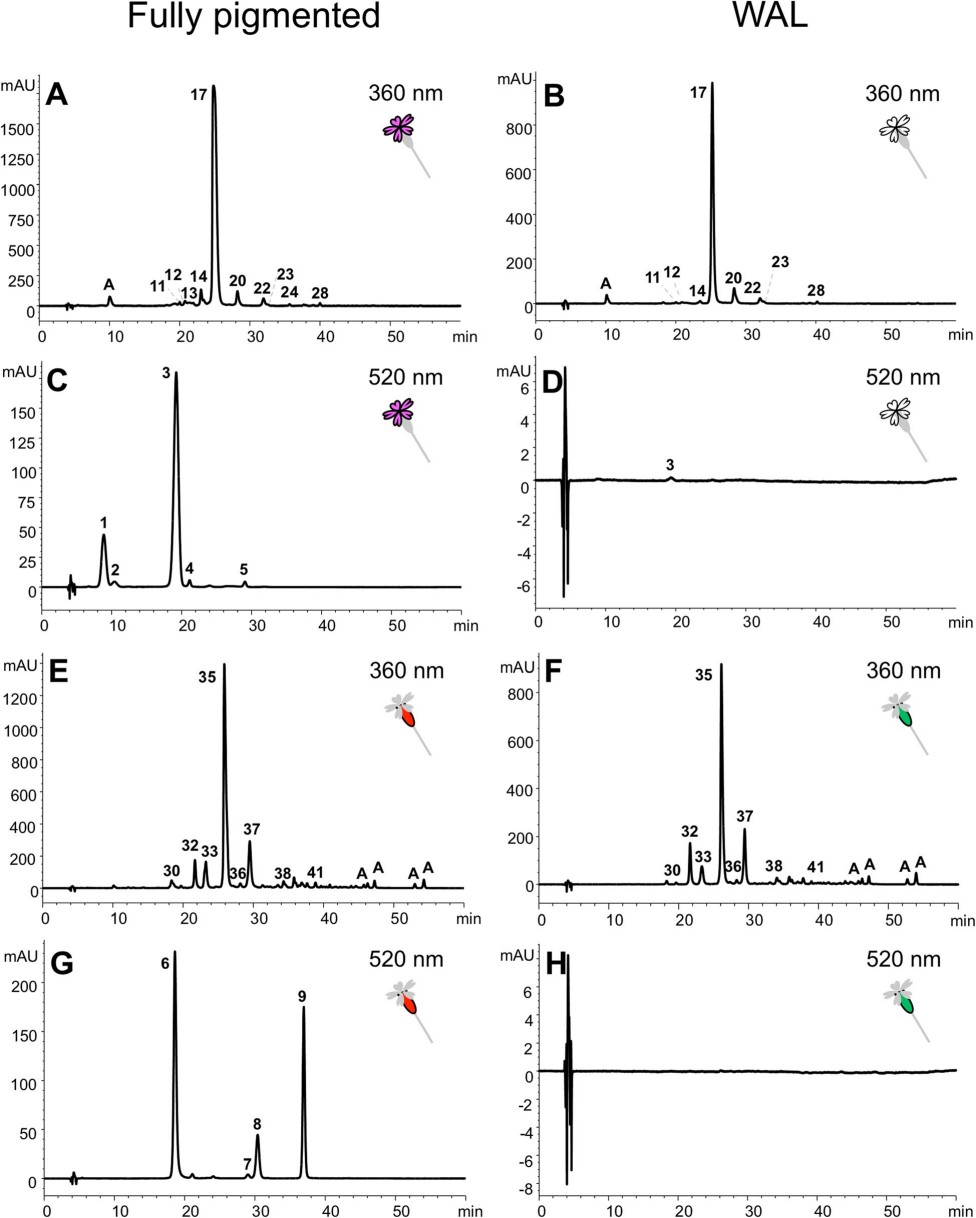
Examples of chromatograms of petals (**a**-**d**) and calyces (**e**-**h**) extracts from fully pigmented and WAL plants from Barra population recorded at 360 nm (flavones) and at 520 nm (anthocyanins). Only main peaks are numbered (details are shown in Additional file 5: Figure S2, Additional file 6: Figure S3 and Additional file 4: Table S3; A = phenolic acids)

Five anthocyanins and 21 flavones were identified in petals, as well as four anthocyanins and 19 flavones in photosynthetic tissues (Additional file 4: Table S3). The anthocyanins detected were cyanidin derivatives in all cases, but with different substituents in petals and photosynthetic tissues (Fig. 2 and Additional file 4: Table S3). The main anthocyanin present in pigmented petals was a glycosylated cyanidin with two sugars (one rhamnosylglucose and one glucose) and acylated with acetic acid (representing 71.0–74.1% of the total anthocyanin concentrations; peak 3 in Fig. 2c). In photosynthetic tissues, the structures of the predominant anthocyanins were simpler, with only one sugar attached to the aglycone (78.0–99.4%; peaks 6–9 in Fig. 2g).

The flavone composition was also different in petals compared to photosynthetic tissues: isovitexin derivatives were mostly accumulated in petals whereas isoorientin derivatives were the main flavones present in photosynthetic tissues (Table 1 and Additional file 4: Table S3). The primary petal flavone was an isovitexin glycosylated with two pentose sugars (69.5–88.3% of the total flavone concentrations in the three phenotypes; peak 17 in Fig. 2a and b). An isoorientin derivative containing an additional hexose and a caffeoyl residue was the main flavone present in calyces (58.8–63.2%), leaves (42.9–58.1%) and stems (50.6–57.8%; peak 35 in Fig. 2e and f). In all tissues, isoscoparin derivatives were also detected, but at relatively low levels (< 11%; Table 1 and Additional file 4: Table S3).

**Table 1:** Mean concentration (mg g^− 1^ FW; fresh weight) of main groups of anthocyanins and flavones from petals and photosynthetic tissues of fully pigmented (F.P.), PAL and WAL phenotypes of Barra and Breña populations. Flavonoid quantification was performed from the area of the peaks detected in the chromatogram using HPLC-DAD-MS^n^. Four plants per phenotype and population were analyzed. Flavones were grouped according to the functional C-glycoside flavone core, the additional glycoses types and the hydroxycinnamic acid types linked to flavone skeleton. Percentages of each flavonoid groups per total flavones are shown in parentheses under concentration values. Anthocyanins (cyanidin derivatives) showed similar chemical characteristics and were grouped into a single category. “–” indicates that the compound was below the detection level

	Petals					Calyces					Leaves					Stems				
	Barra			Breña		Barra			Breña		Barra		Breña			Barra			Breña	
	F.P.	PAL	WAL	F.P.	WAL	F.P.	PAL	WAL	F.P.	WAL	F.P.	PAL	WAL	F.P.	WAL	F.P.	PAL	WAL	F.P.	WAL
Anthocyanins																				
Total:	1.65	0.03	0.02	2.82	0.07	0.28	0.35	< 0.01	0.23	–	< 0.01	0.02	–	0.05	–	0.29	0.18	–	0.29	–
Flavones																				
Total:	92.5	95.2	100.9	69.5	88.1	7.56	9.23	9.31	9.88	4.38	5.32	4.73	8.76	7.50	5.73	4.11	4.55	4.05	5.42	5.19
C-glycoside flavone core^a^																				
Isovitexin	86.2 (93.1)	90.5 (95.1)	84.2 (83.4)	64.9 (93.3)	78.0 (88.5)	0.07 (0.96)	0.08 (0.83)	0.11 (1.15)	0.23 (2.35)	0.08 (1.91)	-	-	-	-	-	-	-	-	0.10 (1.84)	0.05 (0.90)
Isoscoparin	2.75 (2.98)	1.73 (1.81)	10.7 (10.6)	3.36 (4.84)	4.27 (4.85)	0.14 (1.89)	0.18 (1.97)	0.15 (1.66)	0.30 (3.06)	0.11 (2.57)	0.26 (4.95)	0.36 (7.55)	0.35 (3.96)	0.55 (7.33)	0.33 (5.74)	0.23 (5.71)	0.28 (6.21)	0.26 (6.39)	0.69 (12.7)	0.52 (10.0)
Isoorientin	1.49 (1.61)	0.96 (1.01)	3.53 (3.49)	0.73 (1.05)	3.28 (3.72)	6.62 (87.5)	7.95 (86.1)	8.25 (88.6)	8.47 (85.7)	3.69 (84.1)	4.76 (89.5)	3.68 (77.7)	7.90 (90.1)	6.48 (86.4)	5.01 (87.4)	3.67 (89.2)	3.88 (85.5)	3.49 (86.2)	4.30 (79.4)	4.02 (77.5)
di-C-glycosides	2.10 (2.27)	2.00 (2.10)	2.52 (2.50)	0.53 (0.76)	2.57 (2.92)	0.73 (9.6)	1.02 (11.0)	0.80 (8.6)	0.88 (8.93)	0.50 (11.4)	0.29 (5.5)	0.70 (14.7)	0.52 (5.89)	0.47 (6.27)	0.40 (6.09)	0.21 (5.08)	0.38 (8.32)	0.30 (7.41)	0.33 (6.07)	0.60 (11.5)
Additional glycoses^a^																				
Hexose	0.22 (0.24)	-	0.77 (0.76)	1.54 (2.22)	3.22 (3.66)	7.52 (99.5)	9.17 (99.4)	9.26 (99.5)	9.18 (92.9)	4.00 (91.2)	5.31 (99.7)	4.72 (99.6)	8.73 (99.6)	7.43 (99.0)	5.68 (99.0)	4.08 (99.4)	4.50 (99.1)	4.00 (98.8)	5.38 (99.2)	5.11 (98.4)
Pentose	88.0 (95.1)	91.4 (96.0)	92.5 (91.7)	63.9 (92.0)	77.5 (88.0)	-	-	-	-	-	-	-	-	-	-	-	-	-	-	-
Hexose and pentose	2.04 (2.20)	1.65 (1.73)	3.27 (3.24)	2.98 (4.29)	3.85 (4.37)	-	-	-	-	-	-	-	-	-	-	-	-	-	-	-
None	2.25 (2.44)	2.17 (2.28)	4.36 (4.32)	1.06 (1.53)	3.53 (4.00)	0.04 (0.48)	0.06 (0.63)	0.05 (0.52)	0.70 (7.10)	0.38 (8.77)	0.01 (0.28)	0.02 (0.39)	0.04 (0.41)	0.07 (0.96)	0.06 (0.98)	0.02 (0.60)	0.04 (0.95)	0.05 (1.23)	0.04 (0.80)	0.08 (1.56)
Acylation^a^																				
Acylated	0.74 (0.80)	1.07 (1.12)	1.91 (1.90)	1.58 (2.27)	2.47 (2.80)	6.82 (90.3)	8.08 (87.6)	8.35 (89.7)	8.22 (83.2)	3.55 (81.0)	5.21 (97.9)	4.50 (95.1)	8.59 (98.0)	7.29 (97.1)	5.53 (96.5)	3.90 (95.0)	4.23 (93.0)	3.85 (95.3)	5.31 (97.9)	4.92 (94.8)
Caffeic ac.	0.54 (0.58)	0.89 (0.93)	1.11 (1.10)	0.29 (0.42)	0.52 (0.59)	5.37 (71.1)	6.46 (70.1)	6.80 (73.1)	6.14 (62.1)	2.66 (60.7)	3.71 (69.8)	3.15 (66.5)	6.36 (72.6)	4.50 (60.0)	3.37 (58.7)	3.01 (73.2)	3.28 (72.2)	3.02 (74.7)	2.91 (53.8)	2.65 (51.1)
Ferulic ac.	0.20 (0.22)	0.18 (0.19)	0.81 (0.80)	1.29 (1.85)	1.95 (2.21)	1.38 (18.2)	1.51 (16.3)	1.46 (15.7)	1.48 (15.0)	0.57 (13.0)	1.39 (26.1)	1.16 (24.6)	1.86 (21.3)	2.03 (27.1)	1.35 (23.5)	0.84 (20.5)	0.83 (18.2)	0.74 (18.3)	0.76 (14.0)	0.81 (15.7)
*p*-coumaric ac.	-	-	-	-	-	-	-	-	-	-	-	-	-	-	-	-	-	-	1.15 (21.2)	0.79 (15.2)
Diacylated	-	-	-	-	-	0.07 (0.96)	0.11 (1.22)	0.09 (0.96)	0.61 (6.14)	0.32 (7.35)	0.11 (2.01)	0.19 (3.98)	0.36 (4.16)	0.76 (10.1)	0.82 (14.2)	0.05 (1.28)	0.12 (2.57)	0.09 (2.26)	0.49 (8.96)	0.67 (12.8)
None	91.8 (99.2)	94.2 (98.4)	99.0 (98.1)	67.9 (97.7)	85.7 (97.2)	0.74 (9.74)	1.14 (12.4)	0.96 (10.3)	1.66 (16.8)	0.83 (19.0)	0.11 (2.08)	0.23 (4.93)	0.18 (2.02)	0.22 (2.91)	0.20 (3.51)	0.21 (5.02)	0.32 (7.00)	0.19 (4.72)	0.11 (2.05)	0.27 (5.17)

^a^chemical characteristics of flavonoids are detailed in Additional file 4: Table S3

Flavones were the most abundant flavonoids detected across tissues. In fully pigmented individuals, concentrations of flavones were 14–56× higher than anthocyanins in petals, calyces and stems; leaf tissues showed an ever great bias, producing flavones at rates >100x that of anthocyanins (Table 1). These differences were even more apparent in white petals of PAL plants and anthocyanin-lacking WAL individuals.

### Variation in flavonoid content among phenotypes using HPLC-DAD-MS^n^

The three phenotypes showed significant differences in their anthocyanin concentrations. In petals, PAL and WAL phenotypes accumulated only 1% of the same anthocyanins found in the fully pigmented phenotype (Fig. 2d; Tables 1 and 2). In calyces and stems, WAL phenotypes produced undetectable concentration of anthocyanins (Fig. 2h), whereas fully pigmented and PAL phenotypes showed similar anthocyanin levels. In leaves, anthocyanin concentration was very low and statistically similar for the three phenotypes.

**Table 2:** Results from ANOVAs and MANOVAs comparing the anthocyanin and flavone contents among phenotypes in Barra (fully pigmented, PAL and WAL) and Breña (fully pigmented and WAL). Anthocyanin and flavone concentrations were obtained from HPLC analyses performed in four plants of each phenotype. Comparisons were made independently for each plant tissue. Total anthocyanins were considered for anthocyanin analyses, whereas main groups according to the C-glycoside flavone core were considered for flavone analyses (see Table 1)

	Tissue	Anthocyanins				Flavones			
		ANOVA test				MANOVA test			
		SS	d.f.	*F*	*P*	Wilk’s lambda	*F*	d.f.	*P*
Barra	Petals	6.403	2, 12	19.32	0.001^a^	0.215	1.735	4, 12	0.188
	Calyces	0.272	2, 12	8.249	0.009^b^	0.563	0.500	4, 12	0.834
	Leaves	0.001	2, 12	2.627	0.126	0.256	1.463	4, 12	0.266
	Stems	0.173	2, 12	17.28	< 0.001^b^	0.248	1.510	4, 12	0.251
Breña	Petals	15.11	1, 8	49.89	< 0.001	0.659	0.389	4, 8	0.808
	Calyces	0.106	1, 8	30.76	< 0.001	0.260	2.131	4, 8	0.280
	Leaves	0.004	1, 8	1.121	0.330	0.445	0.935	4, 8	0.544
	Stems	0.170	1, 8	10.71	0.017	0.121	5.448	4, 8	0.098

^a^post hoc Tukey test showed significant differences between fully pigmented vs. PAL and WAL phenotypes (*P* < 0.05)

^b^post hoc Tukey test showed significant differences between WAL vs. fully pigmented and PAL phenotypes (*P* < 0.05)

In contrast to the differences found in anthocyanin concentrations, the three phenotypes showed minimal differences in their flavone content (Fig. 2a, b, e and f), and only three petal flavones (~ 1.5% of total flavones) were not present in all phenotypes (see Additional file 4: Table S3). We found differences in flavone composition between the polymorphic and non-polymorphic populations, with five compounds specific to Breña (compounds 16, 18, 21, 33a and 37a in Additional file 4: Table S3). The first three compounds were rare non-acylated O-glycosyl-C-monoglycoside flavones of petals (< 1% of total flavones), whereas the other two were moderately abundant in photosynthetic tissues (4.17–21.2% of total flavones). Thus, PCA based on the flavone composition and concentration showed higher separation between populations than among phenotypes of each population (Additional file 7: Figure S4). When we compared the flavone concentration of each specific group of flavones (i.e. derivatives of isovitexin, isoorientin and isoscoparin, and di-C-glycosides), we found no significant difference among phenotypes neither in the polymorphic nor the non-polymorphic population (Table 2). Similar results were obtained when using the relative proportion of flavones in MANOVAs (Additional file 8: Table S4).

### Variation in flavonoid content among phenotypes measured spectrophotometrically

**Fig. 3:**
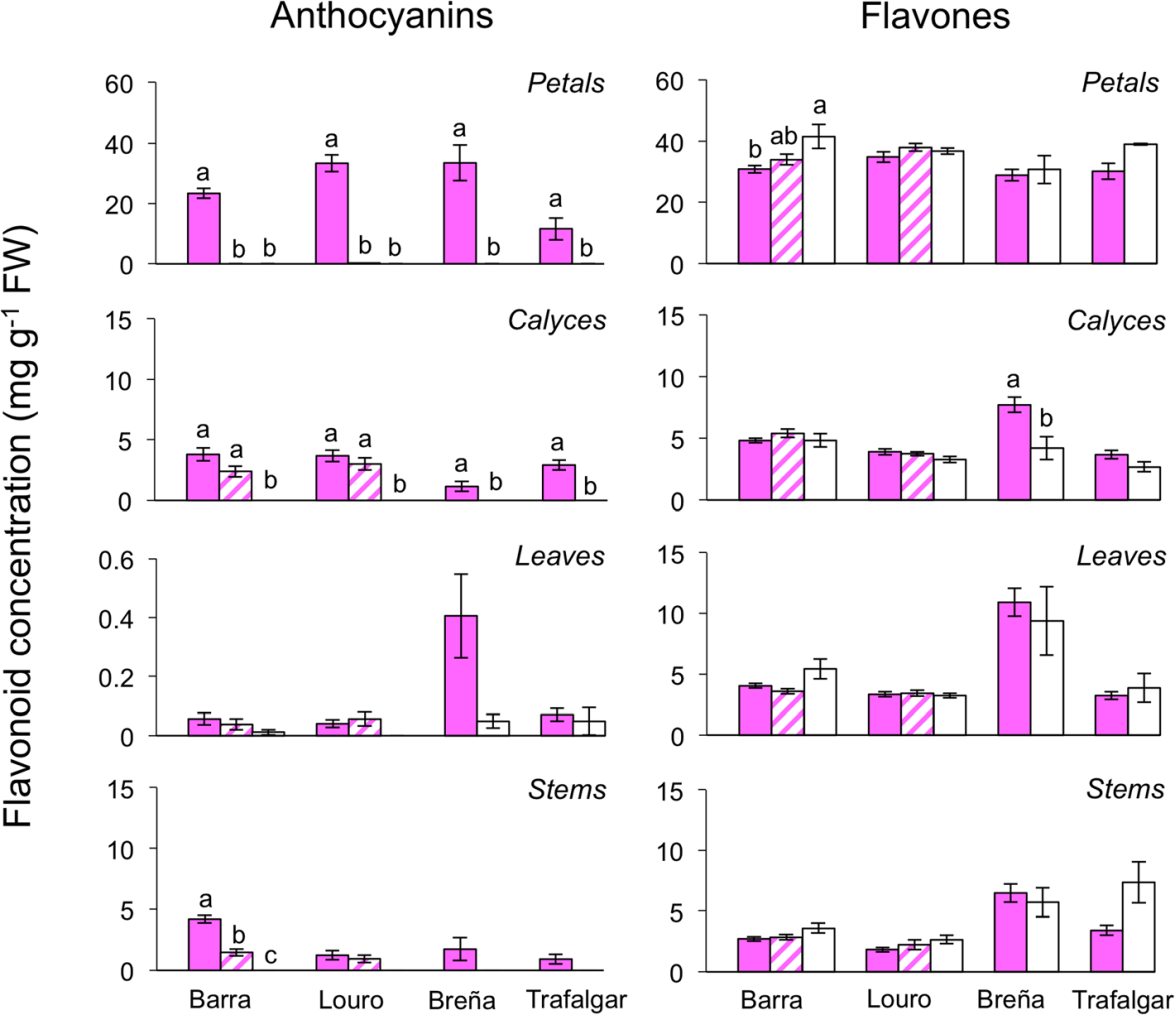
Flavonoid concentrations measured by spectrophotometry in the phenotypes of *S. littorea* from the polymorphic (Barra and Louro) and non-polymorphic (Trafalgar and Breña) petal-color populations. Mean (± s.e.) concentrations of anthocyanins and non-anthocyanin flavonoids in the four studied plant tissues are showed. Pink, pink-white striped and white bars represent fully pigmented, PAL and WAL phenotypes, respectively. Letters indicate significant differences (*P* < 0.05) from post hoc multiple comparisons among phenotypes within each population. Note the different scale between plant tissues and flavonoid types. FW, fresh weight

When expanding the sampling to more individuals and populations, spectrophotometric quantification showed similar pattern of anthocyanin and flavone production to that found in the HPLC analysis. In the two polymorphic populations, the three phenotypes showed significant differences for the anthocyanin accumulation in all tissues except for leaves, which showed very low values in all phenotypes (Fig. 3; Table 3). In petals, PAL and WAL phenotypes produced near zero anthocyanin concentration, whereas in photosynthetic tissues only the WAL phenotype lacked anthocyanins. The three phenotypes showed statistically similar flavone concentrations in photosynthetic tissues. In petals, significant differences were found due to the higher flavone content in WAL plants from Barra (Fig. 3). Between populations, significant differences were found for the anthocyanin production in petals and stems, and for the flavone production in all tissues. Anthocyanins and flavones concentrations were, in general, higher in Barra than Louro.

**Table 3:** Results from generalized linear models (GLMs) testing differences among phenotypes, populations and their interaction on the production of total anthocyanins and non-anthocyanin flavonoids in each plant tissue. GLMs were performed separately in polymorphic (Barra and Louro) and non-polymorphic populations (Breña and Trafalgar). Anthocyanin and flavone concentrations were obtained from spectrophotometric quantification of flavonoids

Source of variation	Polymorphic populations						Non-polymorphic populations					
	Anthocyanins			Flavones			Anthocyanins			Flavones		
	d.f.	*F*	*P*	d.f.	*F*	*P*	d.f.	*F*	*P*	d.f.	*F*	*P*
Petals												
Phenotype	2	1447.9	< 0.001	2	4.122	0.020	1	74.58	< 0.001	1	1.284	0.269
Population	1	6.450	0.013	1	4.510	0.036	1	11.54	0.002	1	0.644	0.430
Phen. x Pop.	2	2.525	0.086	2	1.229	0.298	1	2.939	0.100	1	0.945	0.341
Calyces												
Phenotype	2	33.47	< 0.001	2	1.019	0.366	1	27.67	< 0.001	1	10.27	0.004
Population	1	0.368	0.546	1	35.15	< 0.001	1	8.231	0.008	1	20.10	< 0.001
Phen. x Pop.	2	0.314	0.732	2	0.688	0.506	1	3.475	0.075	1	1.785	0.194
Leaves												
Phenotype	2	1.512	0.227	2	1.407	0.251	1	2.864	0.104	1	0.032	0.860
Population	1	0.001	0.990	1	10.48	0.002	1	4.672	0.041	1	35.24	< 0.001
Phen. x Pop.	2	0.422	0.657	2	2.862	0.063	1	1.391	0.250	1	1.366	0.254
Stems												
Phenotype	2	25.28	< 0.001	2	2.285	0.109	1	3.266	0.084	1	1.994	0.172
Population	1	27.78	< 0.001	1	15.49	< 0.001	1	0.157	0.696	1	11.98	0.002
Phen. x Pop.	2	7.131	0.001	2	0.101	0.904	1	0.042	0.840	1	8.106	0.009

In non-polymorphic populations, anthocyanin production in petals and calyces were significantly different between fully pigmented and WAL phenotypes, and near significant in stems (Fig. 3; Table 3). Flavone concentrations in both phenotypes were similar in all tissues except for calyces, in which WAL plants from Breña showed nearly half concentrations compared to fully pigmented plants (Fig. 3). Between populations, significant differences were found for anthocyanins in all tissues except for the stems, and for flavones in all photosynthetic tissues, showing higher concentration levels in Breña population.

## Discussion

In this study, we found that shore campion accumulates both anthocyanins and flavones, but specific classes of these compounds were differentially produced in petals versus photosynthetic calyces, leaves and stems. Fully pigmented and PAL plants showed similar anthocyanin content in the analyzed tissues, except for the obvious absence in petals, whereas WAL phenotype lacks anthocyanins in the whole plant. In contrast, plants with white petals (both PAL and WAL phenotypes) have similar flavone composition and concentration compared to pink-flowered plants. Thus, the synthesis of flavones in each tissue of both PAL and WAL phenotypes seems to be not influenced by the loss of anthocyanins. This pattern of anthocyanin and flavone production in all phenotypes was congruent in the distant polymorphic and non-polymorphic populations. Together, these results suggest that anthocyanin accumulation in photosynthetic tissues are directly or indirectly involved in petal color polymorphism persistence. Below, we discuss these findings in view of the frequency in which PAL and WAL phenotypes are found in natural populations.

**Fig. 4:**
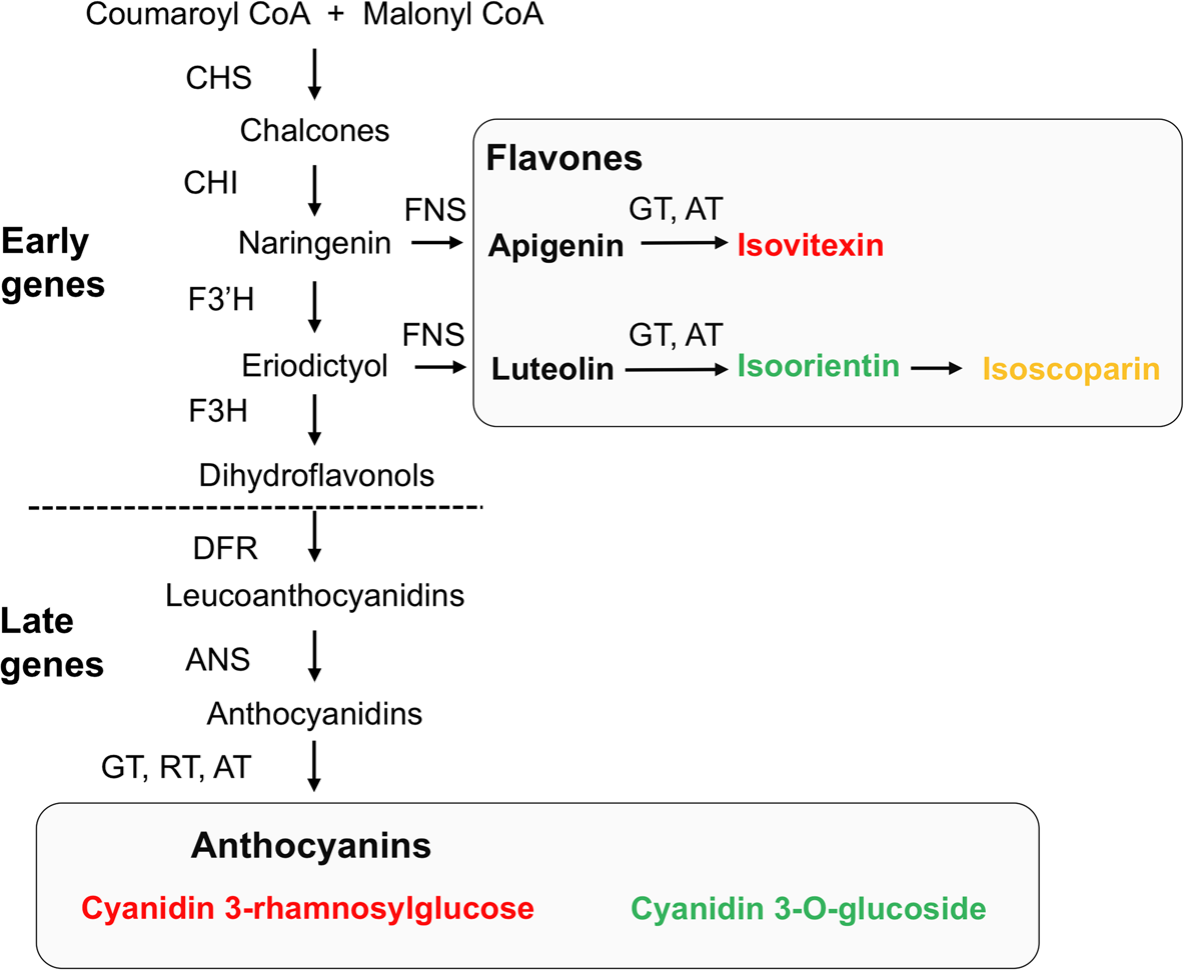
Simplified flavonoid biosynthetic pathway in *Silene littorea*. Enzymatic activities (capital letters next to arrows) and metabolic products are indicated. Main anthocyanins and flavones detected by HPLC-DAD-MS^n^ are in boxes with red, green and yellow letters for compounds found in petals, photosynthetic tissues or both, respectively. The biosynthetic route was divided into early and late halves using a dotted line based on genes involved in the synthesis of upstream (flavones) and downstream (anthocyanins) products of the biosynthetic pathway. CHS: chalcone synthase; CHI, chalcone isomerase; F3’H, flavonoid 3’hydroxylase; FNS, flavone synthase; F3H, flavanone-3-hydroxylase; DFR, dihydroflavonol 4-reductase; ANS, anthocyanidin synthase; GT, glucosyl transferase; RT, rhamnosyl transferase; AT, acyltransferase

One of the most significant findings reported here is that PAL and WAL plants exhibited similar flavone content as fully pigmented plants, even though they lacked anthocyanins in either their petals or petals and vegetative tissues. In a previous study analyzing the sequences and gene expression of ABP genes in petals of *S. littorea*, Casimiro-Soriguer et al. [47] suggested that anthocyanin petal-loss in PAL individuals is caused by a decreased expression of flavanone-3-hydroxylase (*F3h*) controlled by a petal specific regulatory gene, *SlMyb1a*. Detection of flavones in PAL petals is consistent with the blockage of the ABP at *F3 h* since flavones are synthetized from naringenin or eriodictyol, which are produced in the steps immediately preceding F3H (Fig. 4). We suggest that downregulation of *F3 h* prevents a blockage of flavone production [48] and redirects flux from anthocyanins to flavones in white petals of *S. littorea*, as is described in other flower color polymorphic species [49, 50]. Similarly, mutations leading to the complete lack of anthocyanins in WAL individuals may occur later in the pathway to preserve flavone production. In *Mimulus lewisii* and *Iochroma calycinum*, mutations in coding regions of a late gene of the ABP, the dihydroflavonol 4-reductase (*Dfr*), cause the complete loss of anthocyanins of rare white-flowered individuals [51, 52]. A similar downstream blockage of the ABP, but not necessarily caused by inactivation of DFR, could explain the absence of anthocyanins in WAL individuals.

Although several studies have examined the genetic and molecular basis for PAL and WAL phenotypes [11, 29, 48, 51–53], this is the first study that has compared the complete flavonoid profile in these two forms of white-flowered individuals. Nevertheless, some studies have approached this goal in wild species. For example, flowers of the rare white-petal phenotype of *Iochroma calycinum* accumulate the same flavonol (quercetin) that the pigmented phenotype as determined by thin layer chromatography [52]. Although comparisons between natural and horticultural plants should be addressed carefully since they are under different selection when in cultivation, there is some biochemical knowledge available for ornamental plants. Several studies have reported similar flavonol profiles for white-flowered and pigmented lines, as for lisianthus (*Eustoma grandiflorum*) and gentians (*Gentiana triflora*) [54, 55]; however, some white-flowered lines showed different flavonoid profiles, probably because of distinct genetic blockage of the ABP. We have found that all WAL plants of the shore campion showed the same flavonoid profile. This lack of flavonoid diversity in WAL phenotype reinforces the assumption that loss-of-function mutations may target specific late genes rather than the early genes of the ABP, which would compromise flavone production. The shore campion does not produce others classes of flavonoids others than flavones and anthocyanins [47]. Thus, any mutation affecting an ABP gene prior to *F3 h* should preclude any flavone production (Fig. 4), making this mutation selectively disadvantageous due to the decisive role of these compounds to plant development and survival [28, 37, 56].

Loss of anthocyanin pigments is relatively common in nature [39, 40, 57], but its effects on plant fitness will determine the fate of white-flowered individuals. In several species, PAL phenotypes generally show similar, or even higher, fitness than fully pigmented plants ([40, 58, 59]; but see, for instance, [11]), resulting in stable flower color polymorphism in populations. In the shore campion, the pink-white polymorphism is maintained over the years, and white flowers represents 8–21% of total plants in the two polymorphic populations. Myb-mediated loss of anthocyanins, as for *S. littorea*, are frequently cell or tissue specific [60, 61] and allow downregulation of petal anthocyanins without hindering anthocyanin accumulation in other tissues. Our biochemical results confirmed that PAL and fully pigmented plants have similar anthocyanin content in photosynthetic tissues, in addition to have similar flavone content, which is expected to have few or no pleiotropic effects that could alter fitness of PAL phenotypes [17, 34]. In fact, snails and caterpillars produced similar herbivory levels in leaves of fully pigmented and PAL plants of Barra, but white petals of PAL plants showed higher hemipteran florivory that petals of fully pigmented plants (M.L.B. 2019, unpublished data).

A key question arising from our findings is, “Why are WAL phenotypes so rare even if they have similar amounts of protective flavones than fully pigmented plants?”. Flavones share many of the numerous protective biological functions attributed to anthocyanins [27, 56, 62], and are at least 14 times more abundant than anthocyanins across tissues of the shore campion, as commonly found in other species [63]. In *S. littorea*, petal flavones (isovitexins) could be involved in regulation of vacuole homeostasis in epidermal cells and/or act as co-pigments of anthocyanins [64, 65], whereas flavones accumulated in photosynthetic tissues (isoorientins) are effective antioxidants that may play important functions in stress tolerance [28, 62]. Since these protective flavones are accumulated in reproductive and vegetative tissues of WAL plants, it seems plausible that the loss of anthocyanins could be involved in the ecological disadvantage by which WAL phenotype remains scarce in the populations. In fact, it is recently proposed that anthocyanins may play a decisive role in the regulation of signaling cascades responsible for cell growth and differentiation; thus, controlling important developmental processes [27, 28, 66, 67]. In addition, genetic linkage between ABP genes and other loci affecting fitness [16], as well as the metabolic cross-talk between flavonoid and other metabolic pathways [68], are other possible explanations for why loss of anthocyanins (not the flavonoid intermediates) seems to restrict the spread of WAL phenotypes. Taken together, our results suggest that the ability to produce anthocyanin pigments in photosynthetic tissues of the shore campion is associated with the ability to generate stable petal color polymorphisms.

## Conclusions

In summary, our results show striking differences in the ability to synthesize anthocyanins between fully pigmented and white-petal variants of *S. littorea*, whereas flavone production is not affected by loss of anthocyanin pigments. Differences in flavonoid profile between PAL and WAL individuals are based in the absence of anthocyanins in petals or the whole plant, respectively. The low frequency of WAL plants in natural populations leads us to consider the negative ecological consequences of anthocyanin loss, or other putative pleiotropically-linked traits, in photosynthetic tissues and suggests the critical role of these compounds to generate stable flower color polymorphism. It will be interesting to analyze the flavonoid profile in other species with anthocyanin loss individuals either in the petals or in whole plant, and linking this information with their population frequency. These data will provide new insights into the flower color evolution, which also may produce new knowledge on microevolutionary processes.

## Methods

### Study species and frequency of anthocyanin-deficient phenotypes

*Silene littorea* is an annual wild plant that grows in coastal dune ecosystems from the northwestern to the southeastern Iberian Peninsula [47]. Depending on the degree of human disturbance on their habitat, population’s size ranges from approx. 100 (e.g. Algezur, Odiel) to more than 10,000 individuals (e.g. Sines, Barra). This self-compatible species is entomophilous, but may produce up to 20% of fruit and seed set by spontaneous autogamy [69]. Calyces, leaves and stems produce chlorophylls showing photosynthetic activity [70].

To account for the population frequency of PAL and WAL phenotypes, we sampled 21 populations covering the full distribution range of *S. littorea* (Fig. 1). In populations with more than 1000 estimated individuals, frequency estimates were carried out following a random sampling; in populations with lower number of individuals, the whole population was carefully sampled. Plants were visually categorized to each phenotype based on the absence or presence of anthocyanins in petals and photosynthetic tissues. PAL and WAL individuals showed a similar morphology (plant height, number of open flowers, flower size) than the fully pigmented phenotype.

### Sampling for flavonoid analyses

Flavonoid analyses were conducted in two northwestern petal-color polymorphic populations (Barra and Louro) and in two southern non-polymorphic populations (Breña and Trafalgar; Fig. 1). For complete flavonoid identification, we randomly selected four plants of each fully pigmented, PAL and WAL phenotypes in Barra and four plants from each fully pigmented and WAL phenotypes in Breña. For spectrophotometric flavonoids quantification in these four populations, we extended the sampling to 11–20 plants of each phenotype except for the rare WAL individuals, in which six, five, six and three individuals were analyzed (Barra, Louro, Breña and Trafalgar, respectively). Sampling was carried out from March to April 2016, during the early to mid-flowering period of the species (permissions were not necessary to collect these samples). For each plant, we collected four samples: the petals and calyx of one flower, a leaf and stem sections (1 cm length) from the middle region of the stem. Flavonoids were extracted in 1.5 ml of MeOH containing 1% HCl and stored at − 20 °C in the dark, following the procedure described in Del Valle et al. [44]. Voucher specimens from these populations are deposited at the Pablo de Olavide University Herbarium (UPOS-3954, UPOS-8982, UPOS-8983 and UPOS-8984).

### Flavonoid identification and quantification by HPLC-DAD-MS^n^

A volume of 500 μL of methanolic extracts of each plant tissue (i.e. petals, calyces, leaves and stems) was concentrated in a SpeedVac concentrator (Savant ISS110, Thermo Fisher Scientific, NC, USA) after the addition of 100 μL of ultrapure water (Autwomatic, Wasserlab, Barbatáin, Spain). The volume of the aqueous extracts was then adjusted to 250 μL with acidified water (pH = 1.4, HCl). Aqueous extracts were filtered (Clarinert™ Syring Filters, 0.45 μm, Agela Technologies, DE, USA) prior to the HPLC-DAD-MS^n^ analysis. We followed the procedure described in Alcalde-Eon et al. [71, 72], which has provided satisfactory results in the analyses of anthocyanins in other plant materials and also allows the simultaneous detection of anthocyanins and flavones (see results) in a single run. HPLC analyses were carried out in a Hewlett-Packard 1100 series liquid chromatograph (Agilent Technologies, Waldbronn, Germany). Detection was carried out at 360 nm and 520 nm for flavone and anthocyanin analysis, respectively. Spectra were recorded from 220 to 600 nm.

Mass spectrometric analyses were performed in an API 3200 Qtrap equipped with an ESI source and a triple quadrupole-ion trap mass analyzer that was controlled by Analyst v.5.1 software (Applied Biosystems, Darmstadt, Germany). The HPLC system was connected to the mass spectrometer via the UV cell outlet. Positive mode (ESI^+^) and specific conditions were selected to allow the simultaneous detection of anthocyanins and flavones (see Additional file 9: Supplementary methods for details).

Identification of the compounds was done considering their retention times, UV-Vis spectra, *m/z* of the molecular ion (M^+^) for anthocyanins or *m/z* of the protoned ion [M + H]^+^ for flavones, fragment ions and fragmentation patterns of the compounds. These data supplied valuable information concerning the nature of the aglycones and substituents, which was further compared to the features of standards reported in the literature [73] and to those standards (cyanidin 3-O-glucoside, isovitexin (apigenin 6-C-glucoside) and isoorientin (luteolin 6-C-glucoside)) and samples with known composition on barley (*Hordeum vulgare* L. [74]) analyzed in the same conditions. Furthermore, alkaline and acid hydrolyses were performed in all the types of sample extracts (petals and vegetative parts) to verify the presence of acids in the molecules as well as to determine their identity (see Additional file 9: Supplementary methods for details). Some of the major compounds of petals and photosynthetic tissues were isolated and alkaline hydrolysis was also carried out on them.

Anthocyanin quantification was done from the area of the peaks detected in the chromatogram recorded at 520 nm and using a Cyanidin 3-*O*-glucoside (Polyphenols Labs, Sandnes, Norway) calibration curve. Likewise, flavone quantification was done from the area of the peaks detected in the chromatogram recorded at 360 nm. A calibration curve of isovitexin and isoorientin (Extrasynthese, Genay, France) was employed to quantify the flavones present in petals and photosynthetic tissues, respectively.

HPLC results were analyzed separately for flavones and anthocyanins. In each tissue, exploratory analyses of flavone content of the different phenotypes were performed by principal-component analysis (PCA), using concentrations of all compounds identified. We retained those compounds with the highest principal component loadings. In addition, compounds highly correlated were eliminated to overcome co-linearity. PCA was based on the covariance matrix and without rotation of the extracted component [75]. Confirmatory MANOVAs (multivariate analysis of variance) were carried out when differences between populations were detected [75]. MANOVAs were performed grouping the total flavone composition of plants into four functional groups according to the C-glycoside flavone core (i.e. derivatives of isovitexin, isoorientin and isoscoparin, and di-C-glycosides; see Table 1). Since plants from Barra and Breña locations showed differences in their flavone content (see results) and the PAL phenotype is only present in Barra, both populations were analyzed independently. Because only a few anthocyanin compounds were found in the samples and some phenotypes did not produce anthocyanins (see results), exploratory PCAs could not be performed; instead, differences in the total anthocyanin concentrations among phenotypes were analyzed using ANOVAs with post hoc Tukey test. ANOVAs, PCAs and MANOVAs were carried out in SPSS v.22.0 (Armonk, NY, IBM Corp.).

### Spectrophotometric flavonoids quantification

We used a Multiskan GO microplate spectrophotometer (Thermo Fisher Scientific Inc., MA, USA) to quantify global anthocyanin and flavone concentrations in order to expand the study to more individuals and populations. Three replicas of 200 μL were measured for of each tissues of all individuals. Absorbances were read at 350 and 520 nm to determine the concentrations of flavones and anthocyanins respectively [46], and their concentrations were calculated using calibration curves of standards of the main compounds found (i.e. cyanidin 3-O-glucoside, isovitexin and isoorientin) and expressed as cyanidin-3-glucoside, isovitexin and isoorientin equivalents in fresh weight, respectively.

Generalized linear models (GLMs) with Gaussian or gamma error distribution were performed in R v3.4.0 [76] to test for differences in the accumulation of anthocyanins and flavones in each plant tissue; phenotype and population were considered as fixed factors. Previously, we tested the error distributions that generated the smaller deviance in the model, using the Akaike’s Information Criterion. *F*-test for analysis of deviance was used to correct for overdispersion [77]. Multiple post hoc comparisons were performed using the “multcomp” R-package [78]. Separated GLMs were performed for polymorphic and non-polymorphic populations, using post hoc comparisons with Bonferroni adjustment to test for differences among phenotypes within each population.

## Supplementary information

**Additional file 1: Table S1.** Frequency of petal anthocyanin loss (PAL) and whole-plant anthocyanin loss (WAL) individuals in natural populations of species with polymorphism caused by loss of anthocyanins in petal and spontaneous white mutants, respectively.

**Additional file 2: Table S2.** Sites, geographical locations, estimated population size and percentage of petal anthocyanin loss (PAL) and whole-plant anthocyanin loss (WAL) phenotypes of *S. littorea*.

**Additional file 3: Figure S1.** Pictures of white-flowered plants of *S. littorea* in which differences in anthocyanin accumulation in photosynthetic tissues between the petal anthocyanin loss (PAL) and whole-plant anthocyanin loss (WAL) phenotypes are shown.

**Additional file 4: Table S3.** Anthocyanins and flavones identified through HPLC-DAD-MS^n^ from methanolic extracts of petals and photosynthetic tissues (calyces, leaves and stems) of *S. littorea* plants.

**Additional file 5: Figure S2.** Chromatogram of the petal extract of a WAL specimen from Breña population recorded at 360 nm.

**Additional file 6: Figure S3.** Chromatogram of the leaf extract of a fully pigmented specimen from Barra population recorded at 360 nm.

**Additional file 7: Figure S4.** Scatter plot of principal components extracted from PCAs using flavone composition detected in petals, calyces, leaves and stems of *S. littorea* phenotypes through HPLC-DAD-MS^n^.

**Additional file 8: Table S4.** Results from MANOVAs comparing the relative proportion of flavones among phenotypes in Barra (fully pigmented, PAL and WAL) and Breña (fully pigmented and WAL).

**Additional file 9.** Supplementary methods. Details of the mass spectrometry conditions, isolation of flavones, and alkaline and acid hydrolysis.

## Data Availability

The datasets supporting the conclusions of this article are included within the article and its additional files.

## Abbreviations

ABP: Anthocyanin biosynthetic pathway
GLM: Generalized linear models
HPLC-DAD-MS^n^: High-performance liquid chromatography coupled with diode-array detection and electrospray ionization tandem mass spectrometry
PAL: Petal anthocyanin loss
WAL: Whole-plant anthocyanin
