# Supplementary material for: Stability of petal color polymorphism: the significance of anthocyanin accumulation in photosynthetic tissues
Source: BMC Plant Biol. 2019 Nov 14;19:496. doi: 10.1186/s12870-019-2082-6 (PMC6854811; doi:10.1186/s12870-019-2082-6)
Supplement: Supplementary file 1 — Additional file 1: Table S1. Frequency of petal anthocyanin loss (PAL) and whole-plant anthocyanin loss (WAL) individuals in natural populations of species with polymorphism caused by loss of anthocyanins in petal and spontaneous white mutants, respectively. [file 12870_2019_2082_MOESM1_ESM.docx]

| **Table S1.** Frequency of petal anthocyanin loss (PAL) and whole-plant anthocyanin loss (WAL) individuals in natural populations of species with polymorphism caused by loss of anthocyanins in petal and spontaneous white mutants, respectively. In addition, presence of non-anthocyanin flavonoids (flavones, flavonols) in flower and vegetative tissues is indicated when biochemical data is available. | | | | |
| --- | --- | --- | --- | --- |
|  | **White-petal phenotype frequency (%)** | **Non-anthocyanin**  **flavonoids** | | **Reference** |
|  |  | **Flower** | **Vegetative** |  |
| **Species with PAL phenotypes^a^** |  |  |  |  |
| *Bletia patula* (Orchidaceae) | ~ 60 | ? | ? | [1] |
| *Cirsium palustre* (Asteraceae) | 0 - 64 | ? | ? | [2] |
| *Corydalis cava* (Fumarioideae) | 5 - 40 | ? | ? | [3] |
| *Cosmos bipinnatus* (Asteraceae) | 10 - 15 | ? | ? | [4] |
| *Gymnadenia rhellicani* (Orchidaceae) | 10 | + | ? | [5] |
| *Ipomoea purpurea* (Solanaceae) | 0 - 43 | ? | ? | [6] |
| *Linanthus parryae* (Polemoniaceae) | 0 – 100 (mean 78) | ? | ? | [7, 8] |
| *Orchis italica* (Orchidaceae) | 12 | ? | ? | E.N. 2016 (unpublished data) |
| *Parrya nudicaulis* (Brassicaceae) | 0 - 24 | + | + | [9]; E.N. and J.B.W. 2016 (unpublished data) |
| *Phlox pilosa* (Polemoniaceae) | 0 - 100 | ? | ? | [10] |
| *Protea aurea* (Proteaceae) | 70 - 95 | ? | ? | [11, 12] |
| *Silene gallica* (Caryophyllaceae) | 0 - 100 | ? | ? | E.N. 2018 (unpublished data) |
| *Silybum marianum* (Asteraceae) | 12 - 24 | ? | ? | [13]; E.N. 2018 (unpublished data) |
| **Species with WAL phenotypes^b^** |  |  |  |  |
| *Borago officinalis* (Boraginaceae) | < 0.01 | + | + | E.N. and J.C.V. 2016 (unpublished data) |
| *Delphinium nelsonii* (Ranunculaceae) | < 0.1 | ? | ? | [14] |
| *Digitalis* *purpurea* (Plantaginaceae) | < 0.001 | ? | ? | [15, 16] |
| *Echium plantagineum* (Boraginaceae) | < 0.1 | ? | ? | [17]; E.N. 2016 (unpublished data) |
| *Ipomoea purpurea* (Solanaceae) | < 0.005 | ? | ? | [18, 19] |
| *Iochroma calycinum* (Solanaceae) | “extremely rare” | + | - | [20] |
| *Lupinus pilosus* (Leguminosae) | < 1 | ? | ? | [21] |
| *Medicago sativa* (Leguminosae) | “rare” | ? | ? | [22] |
| *Mimulus guttatus* (Phrymaceae) | 0.08 (greenhouse) | ? | ? | [23] |
| *Mimulis lewisii* (Phrymaceae) | “rare” | ? | ? | [24] |
| *Orchis mascula* (Orchidaceae) | < 1.4 | ? | ? | [25] |
| *Phlox drummondii* (Polemoniaceae) | 1 | ? | ? | [26] |
| *Silene dioca* (Caryophyllaceae) | “rare” | ? | ? | [27, 28] |
| ^a^, all polymorphic species showed PAL individuals with anthocyanins in vegetative tissues, except in *Cosmos bipinnatus, Gymnadenia rhellicani* and *Silybum marianum* with no available information. ^b^, all species with spontaneous mutants had WAL individuals with lack of anthocyanins in vegetative tissues, except in *Iochroma calycinum* in which wild type plants also showed lack of anthocyanins in vegetative tissues, and in *Ipomoea purpurea* in which variegated pigmentation was found in flowers, stems and leaves. | | | | |

**References**

1. Ackerman JD, Carromero W. Is reproductive success related to color polymorphism in a deception pollinated tropical terrestrial orchid? Caribb J Sci. 2005;41:234–42.

2. Mogford DJ. Flower colour polymorphism in *Cirsium palustre*. Heredity (Edinb). 1974;33:241–56.

3. Olesen JM, Knudsen JT. Scent profiles of flower color morphs of *Corydalis cava* (Fumariaceae) in relation to foraging behavior of bumblebee queens (*Bombus Terrestris*). Biochem Syst Ecol. 1994;22:231–7.

4. Malerba R, Nattero J. Pollinator response to flower color polymorphism and floral display in a plant with a single-locus floral color polymorphism: consequences for plant reproduction. Ecol Res. 2012;27:377–85.

5. Kellenberger RT, Byers KJRP, De Brito Francisco RM, Staedler YM, LaFountain AM, Schönenberger J, et al. Emergence of a floral colour polymorphism by pollinator-mediated overdominance. Nat Commun. 2019;10:63. doi:10.1038/s41467-018-07936-x.

6. Epperson BK, Clegg MT. Spatial-autocorrelation analysis of flower color polymorphisms within substructured populations of morning glory (*Ipomoea purpurea*). Am Nat. 1986;128:840–58.

7. Epling C, Dobzhansky T. Genetics of natural populations. VI. Microgeographic races in *Linanthus parryae*. Genetics. 1942;27 May:317–32.

8. Schemske DW, Bierzychudek P. Perspective: evolution of flower color in the desert annual *Linanthus parryae*: Wright revisited. Evolution (N Y). 2001;55:1269–82.

9. Dick CA, Buenrostro J, Butler T, Carlson ML, Kliebenstein DJ, Whittall JB. Arctic mustard flower color polymorphism controlled by petal-specific downregulation at the threshold of the anthocyanin biosynthetic pathway. PLoS One. 2011;6:e18230.

10. Levin DA, Kerster HW. Phenotypic dimorphism and populational fitness in *Phlox*. Evolution (N Y). 1970;24:128–34. doi:10.2307/2406719.

11. Carlson JE, Holsinger KE. Natural selection on inflorescence color polymorphisms in wild *Protea* populations: the role of pollinators, seed predators, and intertrait correlations. Am J Bot. 2010;97:934–44.

12. Carlson JE, Holsinger KE. Direct and indirect selection on floral pigmentation by pollinators and seed predators in a color polymorphic South African shrub. Oecologia. 2013;171:905–19.

13. Keasar T, Gerchman Y, Lev-Yadun S. A seven-year study of flower-color polymorphism in a Mediterranean annual plant. Basic Appl Ecol. 2016;17:741–50. doi:10.1016/j.baae.2016.10.003.

14. Waser NM, Price M V. Pollinator choice and stabilizing selection for flower color in *Delphinium nelsonii*. Evolution (N Y). 1981;35:376–90. doi:10.1111/j.1558-5646.1981.tb04896.x.

15. Ernst WHO. Scarcity of flower color polymorphism in field populations of *Digitalis Purpurea* L. Flora. 1987;179:231–9.

16. Warren J, Mackenzie S. Why are all colour combinations not equally represented as flower-colour polymorphisms? New Phytol. 2001;151:237–41.

17. Burdon JJ, Marshall DR, Brown AHD. Demographic and genetic changes in populations of *Echium Plantagineum*. J Ecol. 1983;71:667–79. doi:10.2307/2259584.

18. Epperson BK, Clegg MT. Instability at a flower colour locus in the morning glory. J Hered. 1987;78 November:346–52.

19. Coberly LC, Rausher MD. Analysis of a chalcone synthase mutant in *Ipomoea purpurea* reveals a novel function for flavonoids: amelioration of heat stress. Mol Ecol. 2003;12:1113–24.

20. Coburn RA, Griffin RH, Smith SD. Genetic basis for a rare floral mutant in an andean species of solanaceae. Am J Bot. 2015;102:264–72.

21. Pazy B. Flower-colour polymorphism in *Lupinus pilosus* in Israel. Plant Breed. 1987;99:327–9.

22. Talbert LE, Bingham ET. Genetic characterization of a mutable allele in alfalfa (*Medicago sativa* L.). J Hered. 1989;80:407–10.

23. Twyford AD, Caola AM, Choudhary P, Raina R, Friedman J. Loss of color pigmentation is maintained at high frequency in a monkey flower population. Am Nat. 2018;191:135–45. doi:10.1086/694853.

24. Wu CA, Streisfeld MA, Nutter LI, Cross KA. The genetic basis of a rare flower color polymorphism in *Mimulus lewisii* provides insight into the repeatability of evolution. PLoS One. 2013;8:e81173.

25. Dormont L, Delle-Vedove R, Bessière JM, Hossaert-Mc Key M, Schatz B. Rare white-flowered morphs increase the reproductive success of common purple morphs in a food-deceptive orchid. New Phytol. 2010;185:300–10.

26. Levin DA, Brack ET. Natural selection against white petals in *Phlox*. Evolution (N Y). 1995;49:1017–22. doi:10.1111/j.1558-5646.1995.tb02336.x.

27. Kamsteeg J, Brederode J Van, Küppers FJEM, Nigtevecht G van. Anthocyanins isolated from petals of various genotypes of the red campion (*Silene dioica* (L.) Clairv.). Zeitschrift fur Naturforsch C. 1978;33:475–83.

28. Rahmé J, Suter L, Widmer A, Karrenberg S. Inheritance and reproductive consequences of floral anthocyanin deficiency in *Silene dioica* (Caryophyllaceae). Am J Bot. 2014;101:1388–92.
