# Supplementary material for: Stability of petal color polymorphism: the significance of anthocyanin accumulation in photosynthetic tissues
Source: BMC Plant Biol. 2019 Nov 14;19:496. doi: 10.1186/s12870-019-2082-6 (PMC6854811; doi:10.1186/s12870-019-2082-6)
Supplement: Supplementary file 2 — Additional file 2: Table S2. Sites, geographical locations, estimated population size and percentage of petal anthocyanin loss (PAL) and whole-plant anthocyanin loss (WAL) phenotypes of S. littorea. [file 12870_2019_2082_MOESM2_ESM.docx]

| **Table S2.** Sites, geographical locations, estimated population size and percentage of petal anthocyanin loss (PAL) and whole-plant anthocyanin loss (WAL) phenotypes of *S. littorea*. Populations were ordered from NW to SE, covering the full distribution range of the species. | | | | | | | | | | | | | |
| --- | --- | --- | --- | --- | --- | --- | --- | --- | --- | --- | --- | --- | --- |
| **Locality** | **Latitude** | **Longitude** | **Estimated population size** | **2010** | | **2013** | | **2014** | | **2016** | | **2018** | |
|  |  |  |  | PAL | WAL | PAL | WAL | PAL | WAL | PAL | WAL | PAL | WAL |
| Balarés, A Coruña, Spain | 43º 14' 30'' N | 8º 56' 27'' W | 10^3^-10^4^ | - | - | 0 | 0.08 | - | - | - | - | - | - |
| Trece, A Coruña, Spain | 43º 10' 58'' N | 9º 09' 22'' W | 10^2^-10^3^ | - | - | 0 | 0 | - | - | - | - | - | - |
| Lira, A Coruña, Spain | 42º 48' 18'' N | 9º 08' 03'' W | 10^2^-10^3^ | - | - | 0 | 0 | - | - | - | - | - | - |
| Louro, A Coruña, Spain | 42º 46' 17'' N | 9º 07' 29'' W | 10^2^-10^3^ | - | - | 21 | 0.25 | 9 | 0.40 | 8 | 0.05 | - | - |
| Furnas, A Coruña, Spain | 42º 38' 16'' N | 9º 02' 21'' W | 10^3^-10^4^ | - | - | 0 | 0 | - | - | - | - | - | - |
| Areabrava, Pontevedra, Spain | 42º 17' 27'' N | 8º 50' 40'' W | 10^3^-10^4^ | - | - | - | - | 0 | 0 | - | - | - | - |
| Barra, Pontevedra, Spain | 42º 15' 35'' N | 8º 50' 25'' W | 10^4^-10^5^ | - | - | 20 | 0.05 | 14 | 0 | 17 | 0.11 | - | - |
| Miramar, Aveiro, Portugal | 41º 04' 11'' N | 8º 39' 24'' W | 10^2^-10^3^ | - | - | 0 | 0 | - | - | - | - | - | - |
| Nazaré, Leiria, Portugal | 39º 36' 50'' N | 9º 04' 59'' W | 10^2^-10^3^ | - | - | 0 | 0 | - | - | - | - | - | - |
| Cascais, Lisboa, Portugal | 38º 41' 49'' N | 9º 27' 45'' W | 10^2^-10^3^ | - | - | 0 | 0 | 0 | 0 | - | - | - | - |
| Alcácer do Sal, Setúbal, Portugal | 38º 29' 11'' N | 8º 54' 13'' W | 10^3^-10^4^ | 0 | 0 | - | - | 0 | 0 | 0 | 0.12 | - | - |
| Sines, Setúbal, Portugal | 37º 55' 17'' N | 8º 48' 17'' W | 10^3^-10^4^ | - | - | - | - | 0 | 0 | - | - | - | - |
| Aljezur, Faro, Portugal | 37º 20' 22'' N | 8º 51' 07'' W | 10^2^-10^3^ | 0 | 0 | 0 | 0.29 | 0 | 0 | - | - | - | - |
| Cabo San Vicente, Faro, Portugal | 37º 01' 23'' N | 8º 59' 43'' W | 10^2^-10^3^ | 0 | 0 | 0 | 0 | - | - | - | - | - | - |
| Odiel, Huelva, Spain | 37º 09' 14'' N | 6º 54' 19'' W | 10^2^-10^3^ | 0 | 0 |  | - | 0 | 0.38 | - | - | - | - |
| Trafalgar, Cádiz, Spain | 36º 10' 57'' N | 6º 02' 21'' W | 10^4^-10^5^ | 0 | 0 | 0 | 0.17 | 0 | 0.09 | 0 | 0.35 | - | 0.21 |
| Breña, Cádiz, Spain | 36º 11' 22'' N | 5º 56' 58'' W | 10^2^-10^3^ | 0 | 0 | - | - | 0 | 0.19 | 0 | 0.86 | - | 0.26 |
| Manilva, Málaga, Spain | 36º 19' 57'' N | 5º 14' 21'' W | 10^2^-10^3^ | - | - | 0 | 0 | - | - | - | - | - | - |
| Punta Entinas, Almería, Spain | 36º 42' 47'' N | 2º 12' 58'' W | 10^3^-10^4^ | 0 | 0.25 | 0 | 0 | 0 | 0 | - | - | - | - |
| Cabo de Gata, Almería, Spain | 36º 44' 58'' N | 2º 38' 10'' W | 10^2^-10^3^ | 0 | 0 | - | - | 0 | 0 | - | - | - | - |
| Carboneras, Almería, Spain | 36º 57' 45'' N | 1º 53' 59'' W | 10^2^-10^3^ | - | - | - | - | 0 | 0 | - | - | - | - |
| “-“ represents the absence of information of the frequency of each phenotype for a specific population in a certain year. | | | | | | | | | | | | | |
