# Supplementary material for: Stability of petal color polymorphism: the significance of anthocyanin accumulation in photosynthetic tissues
Source: BMC Plant Biol. 2019 Nov 14;19:496. doi: 10.1186/s12870-019-2082-6 (PMC6854811; doi:10.1186/s12870-019-2082-6)
Supplement: Supplementary file 3 — Additional file 3: Figure S1. Pictures of white-flowered plants of S. littorea in which differences in anthocyanin accumulation in photosynthetic tissues between the petal anthocyanin loss (PAL) and whole-plant anthocyanin loss (WAL) phenotypes are shown. [file 12870_2019_2082_MOESM3_ESM.docx]

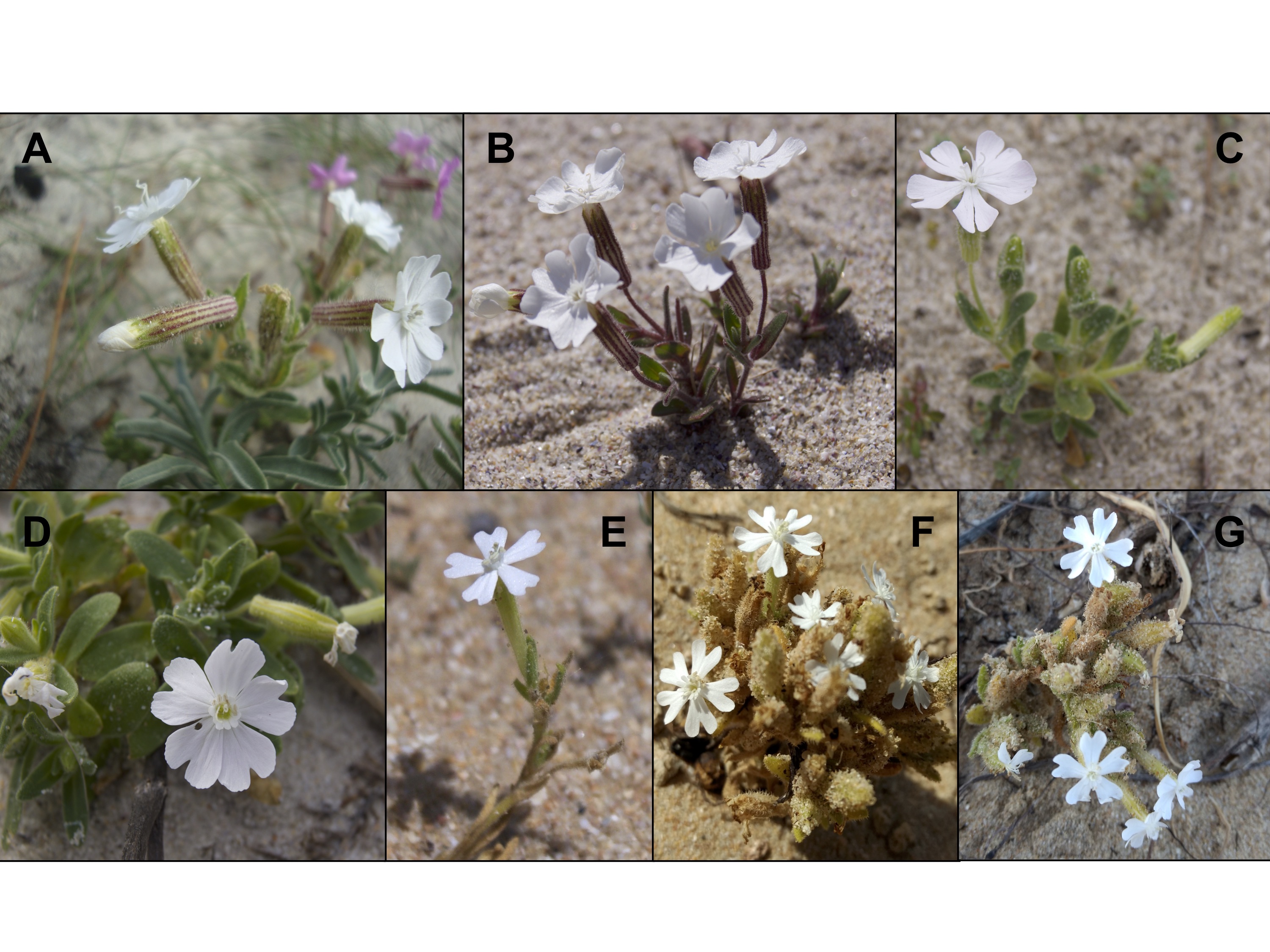


**Figure S1.** Pictures of white-flowered plants of *S. littorea* in which differences in anthocyanin accumulation in photosynthetic tissues between the petal anthocyanin loss (PAL; pictures A and B) and whole-plant anthocyanin loss (WAL; pictures C-G) phenotypes are shown. Photos were taken during winter‐spring 2014-2018 in the following populations: Barra (A-C), Louro (D), Breña (E) and Trafalgar (F, G).
