# Supplementary material for: Stability of petal color polymorphism: the significance of anthocyanin accumulation in photosynthetic tissues
Source: BMC Plant Biol. 2019 Nov 14;19:496. doi: 10.1186/s12870-019-2082-6 (PMC6854811; doi:10.1186/s12870-019-2082-6)
Supplement: Supplementary file 4 — Additional file 4: Table S3. Anthocyanins and flavones identified through HPLC-DAD-MSn from methanolic extracts of petals and photosynthetic tissues (calyces, leaves and stems) of S. littorea plants. [file 12870_2019_2082_MOESM4_ESM.docx]

| **Table S3.** Anthocyanins and flavones identified through HPLC-DAD-MS^n^ from methanolic extracts of petals and photosynthetic tissues (calyces, leaves and stems) of *S. littorea* plants. Two compounds that co-eluted at the same retention time (RT) were named with the same number followed by a different letter. | | | | | | | | | |
| --- | --- | --- | --- | --- | --- | --- | --- | --- | --- |
| **Anthocyanins**  **(520 nm)** | **Peak** | **Rt (min)** | **λ_max_ (nm)** | **M^+^ (*m/z*)** | **Fragment ions in MS^2^ (*m/z*)** | **Aglycone** | **Additional sugar(s)** | **Acylation** | **Type of compounds** |
| Petals | 1 | 9.5 | 278, 516 | 757 | 595,449,287 | Cyanidin | Rhamnosylglucose and glucose | None | 3,5-*O*-triglycosides |
|  | 2 | 11.2 | 277.6, 514 | 611 | 287 | Cyanidin | Glucose (2) | None | 3,5-*O*-diglycosides |
|  | 3 | 19.9 | 277.2, 518.8 | 799 | 637,287,449 | Cyanidin | Rhamnosylglucose and glucose | Acetic ac. | 3,5-*O*-triglycosides acylated |
|  | 4 | 21.4 | 277.6, 440, 516.4 | 595 | 287 | Cyanidin | Rhamnosylglucose | None | 3-*O*-diglycosides |
|  | 5 | 29.5 | 276, 440, 518.8 | 637 | 287 | Cyanidin | Rhamnosylglucose | Acetic ac. | 3-*O*-diglycosides acylated |
| Photosynthetic tissues | 6 | 18.8 | 280, 440, 515.6 | 449 | 287 | Cyanidin | Glucose | None | 3-*O*-monoglycosides |
|  | 7^1^ | 29.5 | 440, 518.8 | 637 | 287 | Cyanidin | Rhamnosylglucose | Acetic ac. | 3-*O*-diglycosides acylated |
|  | 8 | 30.8 | 280.4, 440, 518 | 535 | 287 | Cyanidin | Glucose | Malonic ac. | 3-*O*-monoglycosides acylated |
|  | 9 | 37.5 | 280.4, 440, 518.4 | 549 | 287 | Cyanidin | Glucose | Succinic ac. | 3-*O*-monoglycosides acylated |
|  |  |  |  |  |  |  |  |  |  |
| **Flavones**  **(360 nm)** | **Peak** | **Rt (min)** | **λ_max_ (nm)** | **[M+H]^+^ (*m/z*)** | **Fragment ions in MS^2^ (*m/z*)** | **C-glycosidic core** | **Additional sugar(s)** | **Acylation** | **Type of compounds** |
| Petals | 10 | 19.3 | 342 | 625 | 367,313,343 | Scoparin | Hexose | None | C-monoglycoside O-glycosides |
|  | 11 | 19.8 | 269.2, 340 | 757 | 625,313,343,463,608 | Isoscoparin | Hexose and pentose | None | C-monoglycoside O-glycosides |
|  | 12 | 20.3 | 254, 270.4, 350 | 713 | 449,383,581 | Isoorientin | Pentose (2) | None | C-monoglycoside O-glycosides |
|  | 13a | 21.5 | 269.6, 293.6, 336 | 727 | 433,313,283,337,416 | Isovitexin | Hexose and pentose | None | C-monoglycoside O-glycosides |
|  | 13b | 21.7 | 270.8, 294, 331 | 565 | 325,337,409,427 | Chrysoeriol 6,8-C-dipentoside | None | None | di-C-diglycosides |
|  | 14 | 23.2 | 270.8, 337.6 | 565 | 379,337,469,530,325 | Apigenin 6-C-hexoside-8-C-pentoside | None | None | di-C-diglycosides |
|  | 15 | 23.7 | 257.2, 292.4, 349.2 | 581 | 329,383,431,449 | Isoorientin | Pentose | None | C-monoglycoside O-glycosides |
|  | 16^4^ | 23.9 | 270, 337.2 | 727 | 433,415,313,445,595 | Isovitexin | Hexose and pentose | None | C-monoglycoside O-glycosides |
|  | 17 | 25.0 | 270, 335.6 | 697 | 433,415,565,547,313 | Isovitexin | Pentose (2) | None | C-monoglycoside O-glycosides |
|  | 18^4^ | 26.2 | 269.6, 338 | 595 | 433,415,337,313 | Isovitexin | Hexose | None | C-monoglycoside O-glycosides |
|  | 19 | 26.7 | n.a. | 565 | 397,313,337,433,415,379 | Isovitexin | Pentose | None | C-monoglycoside O-glycosides |
|  | 20a | 28.1 | 270, 334 | 565 | 313,337,433,415,379 | Isovitexin | Pentose | None | C-monoglycoside O-glycosides |
|  | 20b | 28.3 | 270, 334 | 433 | 283,313,337,415,379 | Isovitexin | None | None | C-monoglycoside |
|  | 21^4^ | 29.2 | 254.8, 270.4, 290, 347.6 | 625 | 343,445,463,313,367,397 | Isoscoparin | Hexose | None | C-monoglycoside O-glycosides |
|  | 22 | 31.7 | 254.4, 270.4, 346.8 | 595 | 343,427,313,367,445,463 | Isoscoparin | Pentose | None | C-monoglycoside O-glycosides |
|  | 23 | 32.1 | 254.4, 270.4, 346.9 | 463 | 313,445,409,343,367 | Isoscoparin | None | None | C-monoglycoside |
|  | 24 | 35.6 | 272.8, 328.8 | 903 | 313,177,415,771 | Isovitexin | Hexose and pentose | Ferulic ac. | C-monoglycoside O-glycosides acylated |
|  | 25 | 37.3 | 336.4 | 801 | 177,367,463,487 | Isoscoparin | Hexose | Ferulic ac. | C-monoglycoside O-glycosides acylated |
|  | 26 | 37.7 | 271.6, 292, 300, 331.6 | 771 | 177,313,625,339,397,463 | Isoscoparin | Pentose | Ferulic ac. | C-monoglycoside O-glycosides acylated |
|  | 27 | 39.1 | n.a. | 727 | 415,337,283 | Isovitexin | Pentose | Caffeic ac. | C-monoglycoside O-glycosides acylated |
|  | 28 | 40.1 | 271.6, 256.4, 335.6 | 757 | 295,163,427,343,463,367 | Isoscoparin | Pentose | Caffeic ac. | C-monoglycoside O-glycosides acylated |
| Photosynthetic tissues | 29^2^ | 16.1 | 258.8, 269.6, 350 | 773 | 611,449,431,413,329 | Isoorientin | Hexose (2) | None | C-monoglycoside O-glycosides |
|  | 30 | 18.3 | 271.2, 342 | 773 | 611,329,546,593 | Luteolin 6,8-C-dihexoside | Hexose | None | di-C-glycosides O-glycosides |
|  | 31 | 19.6 | 260.4, 270.4, 350 | 581 | 515,311,461,443,431,421 | Luteolin 6-C-hexoside-8-C-pentoside | None | None | di-C-glycosides |
|  | 32a^2^ | 21.4 | 252.4, 271.6, 337.6 | 935 | 773,449,163,299,329,431 | Isoorientin | Hexose (2) | Caffeic ac. | C-monoglycoside O-glycosides acylated |
|  | 32b | 21.6 | 257.2, 269.6, 292, 348 | 611 | 413,431,329,449,353 | Isoorientin | Hexose | None | C-monoglycoside O-glycosides |
|  | 33a^34^ | 23.1 | 271.2, 290, 300, 335.6 | 565 | 529,511,499,469,427,295 | Apigenin 6-C-hexoside-8-C-pentoside | None | None | di-C-glycosides |
|  | 33b | 23.2 | 273.2, 292.8, 333.6 | 935 | 611,461,353,491,515,163 | Luteolin 6,8-di-C-hexoside | Hexose | Caffeic ac. | di-C-glycoside O-glycosides acylated |
|  | 34^2^ | 25.1 | 252.8, 272, 335.6 | 949 | 787,593,177,491,449,353 | Isoorientin | Hexose (2) | Ferulic ac. | C-monoglycoside O-glycosides acylated |
|  | 35a | 25.7 | 272.4, 292.4, 334 | 773 | 449,329,431,413,287,353 | Isoorientin | Hexose | Caffeic ac. | C-monoglycoside O-glycosides acylated |
|  | 35b | 25.7 | 273.2, 292.4, 333.6 | 949 | 611,449,491,773,515 | Luteolin 6,8-di-C-hexoside | Hexose | Ferulic ac. | di-C-glycoside O-glycosides acylated |
|  | 36 | 27.9 | 272.4, 292, 326.8 | 773 | 383,163,299,329,449,611 | Isoorientin | Hexose | Caffeic ac. | C-monoglycoside O-glycosides acylated |
|  | 37a^4^ | 29.1 | 272.4, 292.4, 326.4 | 757 | 595,449,383,299 | Isoorientin | Hexose | *p*-coumaric ac. | C-monoglycoside O-glycosides acylated |
|  | 37b | 29.3 | 272, 292.4, 334 | 787 | 449,177,431,329,353,299 | Isoorientin | Hexose | Ferulic ac. | C-monoglycoside O-glycosides acylated |
|  | 38a | 34.5 | n.a. | 757 | 433,163,337,325,283 | Isovitexin | Hexose | Caffeic ac. | C-monoglycoside O-glycosides acylated |
|  | 38b | 34.5 | n.a. | 787 | 463,163,445,625 | Isoscoparin | Hexose | Caffeic ac. | C-monoglycoside O-glycosides acylated |
|  | 39 | 37.3 | n.a. | 801 | n.a. | Isoscoparin | Hexose | Ferulic ac. | C-monoglycoside O-glycosides acylated |
|  | 40^2^ | 37.7 | 272.8, 334.8 | 771 | n.a. | Isovitexin | Hexose | Ferulic ac. | C-monoglycoside O-glycosides acylated |
|  | 41 | 39.4 | 278.8, 292.4, 332.4 | 1111 | 787,769,991,667,353,299,637 | Isoorientin | Hexose (2) | Caffeic ac. (2) | C-monoglycoside O-glycosides diacylated |
|  | 42 | 40.5 | 279.2, 292.4, 330.8 | 1125 | 787,769,177,667,625,365,325 | Isoorientin | Hexose (2) | Ferulic ac. (2) | C-monoglycoside O-glycosides diacylated |
| ^1^ not present in leaves; ^2^ not present in calyces; ^3^ not present in leaves and stems; ^4^ exclusive of Breña population; n.a., not available | | | | | | | | | |
