# Supplementary material for: Stability of petal color polymorphism: the significance of anthocyanin accumulation in photosynthetic tissues
Source: BMC Plant Biol. 2019 Nov 14;19:496. doi: 10.1186/s12870-019-2082-6 (PMC6854811; doi:10.1186/s12870-019-2082-6)
Supplement: Supplementary file 6 — Additional file 6: Figure S3. Chromatogram of the leaf extract of a fully pigmented specimen from Barra population recorded at 360 nm. [file 12870_2019_2082_MOESM6_ESM.docx]

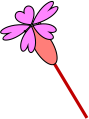

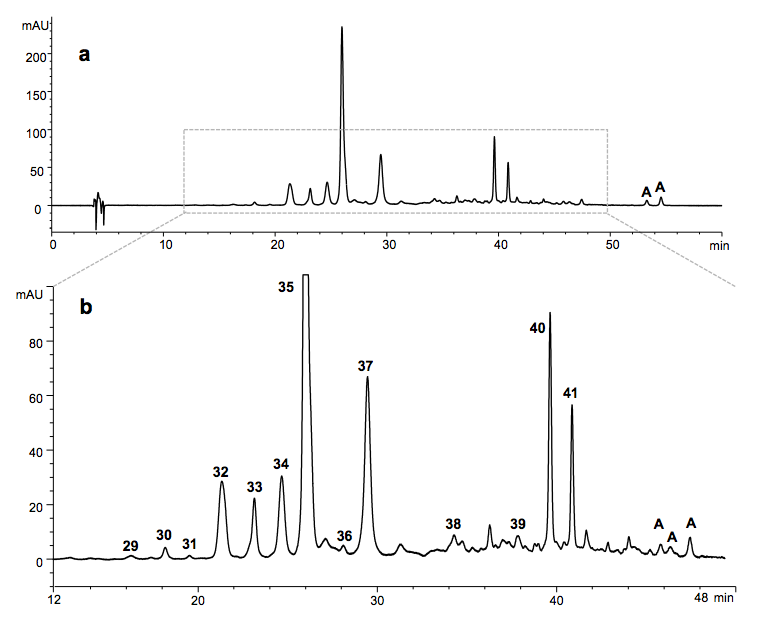


**Figure S3.** Chromatogram of the leaf extract of a fully pigmented specimen from Barra population recorded at 360 nm (A). Zoom on the time range 12-50 min (B). See Additional file 4: Table S3 for more details on compound identities (A = phenolic acids).
