# Supplementary material for: Stability of petal color polymorphism: the significance of anthocyanin accumulation in photosynthetic tissues
Source: BMC Plant Biol. 2019 Nov 14;19:496. doi: 10.1186/s12870-019-2082-6 (PMC6854811; doi:10.1186/s12870-019-2082-6)
Supplement: Supplementary file 7 — Additional file 7: Figure S4. Scatter plot of principal components extracted from PCAs using flavone composition detected in petals, calyces, leaves and stems of S. littorea phenotypes through HPLC-DAD-MSn. [file 12870_2019_2082_MOESM7_ESM.docx]

**Figure S4.** Scatter plot of principal components extracted from PCAs using flavone composition detected in petals (A), calyces (B), leaves (C) and stems (D) of *S. littorea* phenotypes through HPLC-DAD-MS^n^. PCAs were performed for samples obtained from Barra (circles) and Breña (triangles). Pink, grey and green colors were used to represent the fully pigmented, petal anthocyanin loss (PAL) and whole-plant anthocyanin loss (WAL) phenotypes, respectively. Variance explained by each two first principal components is indicated.
