# Supplementary material for: Stability of petal color polymorphism: the significance of anthocyanin accumulation in photosynthetic tissues
Source: BMC Plant Biol. 2019 Nov 14;19:496. doi: 10.1186/s12870-019-2082-6 (PMC6854811; doi:10.1186/s12870-019-2082-6)
Supplement: Supplementary file 8 — Additional file 8: Table S4. Results from MANOVAs comparing the relative proportion of flavones among phenotypes in Barra (fully pigmented, PAL and WAL) and Breña (fully pigmented and WAL). [file 12870_2019_2082_MOESM8_ESM.docx]

| **Table S4.** Results from MANOVAs comparing the relative proportion of flavones among phenotypes in Barra (fully pigmented, PAL and WAL) and Breña (fully pigmented and WAL). The relative proportion of flavones was estimated from flavone concentrations detected in HPLC analyses performed in four plants of each phenotype (see Table 2). Comparisons were made independently for each plant tissue. Main groups according to the C-glycoside flavone core were considered for MANOVAs analyses (see Table 1). | | | | | |
| --- | --- | --- | --- | --- | --- |
|  | **Tissue** | **Wilk's**  **lambda** | ***F*** | **d.f.** | ***P*** |
| Barra | Petals | 0.267 | 2.182 | 4, 12 | 0.107 |
|  | Calyces | 0.585 | 0.461 | 4, 12 | 0.861 |
|  | Leaves | 0.249 | 2.338 | 4, 12 | 0.089 |
|  | Stems | 0.146 | 2.420 | 4, 12 | 0.081 |
|  |  |  |  |  |  |
| Breña | Petals | 0.630 | 0.783 | 4, 8 | 0.562 |
|  | Calyces | 0.417 | 1.863 | 4, 8 | 0.277 |
|  | Leaves | 0.478 | 1.457 | 4, 8 | 0.352 |
|  | Stems | 0.213 | 4.915 | 4, 8 | 0.079 |
