# Supplementary material for: Stability of petal color polymorphism: the significance of anthocyanin accumulation in photosynthetic tissues
Source: BMC Plant Biol. 2019 Nov 14;19:496. doi: 10.1186/s12870-019-2082-6 (PMC6854811; doi:10.1186/s12870-019-2082-6)
Supplement: Supplementary file 9 — Additional file 9. Supplementary methods. Details of the mass spectrometry conditions, isolation of flavones, and alkaline and acid hydrolysis. [file 12870_2019_2082_MOESM9_ESM.docx]

**Supplementary methods:**

**Mass spectrometry conditions**

Zero grade air served as nebulizer (GS1) and turbo gas (GS2) for solvent drying. Nitrogen served as curtain (CUR) and collision gas (CAD). The ionization and fragmentation conditions were optimized by direct infusion of a solution of malvidin 3-*O*-glucoside. GS1 and GS2 were set at 40 psi and 50 psi, respectively. CUR was set at 10 psi and CAD was set as “high”. Declustering potential (DP) was set at 20 V, entrance potential (EP) at 10 V, ion spray voltage (IS) at 5000 V, and the temperature of the probe (TEM) at 600ºC. Both quadrupoles were set at unit resolution. Three types of mass experiments were performed: full mass analysis (EMS mode, collision energy (CE) 10 V), where all the ions were detected, MS^2^ analysis (EPI mode, CE 30 V), where the major ion of the full mass analysis was fragmented, and MS^3^ analysis (CE 30 V, excitation energy (AF2) 80 V), where the major fragment ion of the MS^2^ analysis was, in turn, fragmented. Spectra were recorded between *m/z* 150 and 1400.

**Isolation of flavones**

The same equipment as that employed in the HPLC-DAD analyses was used for the isolation of certain compounds. The chromatographic method was previously optimized and used in our laboratory for the isolation of flavonoids in other plant samples [1]. Each selected sample was concentrated at the head of the column (20 consecutive injections of 100 μL of sample with isocratic 100% ultrapure water, solvent C) and then eluted by a gradient between A, B and C solvents [1]. Spectra were recorded from 220 to 600 nm. Isolated compounds were collected through the UV-cell outlet.

**Alkaline hydrolysis**

Alkaline hydrolysis was performed in the extracts obtained from petals and from vegetative parts in order to establish the peaks that contained acylation and to determine the identities of the acids. It was also performed in some of the major compounds of the extracts that were previously isolated. The methodology was the same as that previously employed in our laboratory for the alkaline hydrolysis of anthocyanins in other plant material [1], but slightly modified [2] to avoid degradation of dihydroxy phenolic acids, such as caffeic acid. To be precise the alkaline hydrolysis was carried out in the presence of ascorbic acid (1%) and ethylenediaminetetraacetic acid (EDTA) 10 mM, which were initially added to the samples. Then, each sample was introduced in a screw-cap test tube, 10% aqueous KOH was added until alkaline pH (pH indicator paper roll), nitrogen was sparged into the solution and the tube was capped and maintained in the dark at room temperature for 10 minutes. The solution was neutralised with 3N HCl until acid pH. The organic phase, containing the phenolic acids, and the aqueous phase, containing the deacylated compounds, were separated with ethyl ether, concentrated under vacuum (SpeedVac) and re-dissolved either in MeOH:H_2_O (10:90) (phenolic acids) or in acidified water (pH=1.4, HCl) (deacylated flavonoids). Both phases were analysed by HPLC-DAD-MS^n^ with the same method as described in the methods section.

**Acid hydrolysis**

Ten mL of 3N HCl was added to the aqueous phase resulting from the alkaline hydrolysis in a screw-cap test tube. Then, nitrogen was sparged and the test tube was capped. The tube was placed at 100ºC for 60 min and then cooled in an ice bath. The hydrolysate was purified using a Waters C-18 Sep-Pak® cartridge previously activated with methanol:HCl 0.1N (95:5) and equilibrated with ultrapure water. The sample was loaded onto the cartridge and ultrapure water was first added in order to elute the sugars released from the flavonoids. Then, the aglycones and C-glycosidic cores were eluted with acidified methanol. This fraction was concentrated under vacuum and then analyzed by HPLC-DAD-MS^n^.

**References**

1. Alcalde-Eon C, Rivas-Gonzalo JC, Muñoz O, Escribano-Bailón MT. *Schizanthus grahamii* and *Schizanthus hookeri*. Is there any relationship between their anthocyanin compositions and their different pollination syndromes? Phytochemistry. 2013;85:62–71. doi:10.1016/j.phytochem.2012.09.013.

2. Nardini M, Cirillo E, Natella F, Mencarelli D, Comisso A, Scaccini C. Detection of bound phenolic acids: prevention by ascorbic acid and ethylenediaminetetraacetic acid of degradation of phenolic acids during alkaline hydrolysis. Food Chem. 2002;79:119–24.
